# Food-Associated Stress Primes Foodborne Pathogens for the Gastrointestinal Phase of Infection

**DOI:** 10.3389/fmicb.2018.01962

**Published:** 2018-08-23

**Authors:** Nathan Horn, Arun K. Bhunia

**Affiliations:** ^1^Department of Animal Sciences, Purdue University, West Lafayette, IN, United States; ^2^Molecular Food Microbiology Laboratory, Department of Food Science, Purdue University, West Lafayette, IN, United States; ^3^Department of Comparative Pathobiology, Purdue University, West Lafayette, IN, United States

**Keywords:** human pathogen, food processing, pathogenesis, infection, stress response and adaptation, pathogen survival, gut, immunity

## Abstract

The incidence of foodborne outbreaks and product recalls is on the rise. The ability of the pathogen to adapt and survive under stressful environments of food processing and the host gastrointestinal tract may contribute to increasing foodborne illnesses. In the host, multiple factors such as bacteriolytic enzymes, acidic pH, bile, resident microflora, antimicrobial peptides, and innate and adaptive immune responses are essential in eliminating pathogens. Likewise, food processing and preservation techniques are employed to eliminate or reduce human pathogens load in food. However, sub-lethal processing or preservation treatments may evoke bacterial coping mechanisms that alter gene expression, specifically and broadly, resulting in resistance to the bactericidal insults. Furthermore, environmentally cued changes in gene expression can lead to changes in bacterial adhesion, colonization, invasion, and toxin production that contribute to pathogen virulence. The shared microenvironment between the food preservation techniques and the host gastrointestinal tract drives microbes to adapt to the stressful environment, resulting in enhanced virulence and infectivity during a foodborne illness episode.

## Introduction

Foodborne illnesses cause considerable morbidity, mortality, and economic losses globally. The World Health Organization (WHO) estimates approximately 2 billion illnesses resulting in over 1 million deaths caused by 22 major foodborne pathogens (Kirk et al., 2015). The European Food Safety Authority (EFSA) reported an upward trend of foodborne outbreaks (5,196) in 2013 in 28 member states and 4 non-member states (EFSA, 2015). In the United States of America, the incidence of food-related disease outbreaks and product recalls are on the rise (Murphree et al., 2012; Gould et al., 2013). The foodborne illness is blamed for approximately 48 million cases, 128,000 hospitalizations, 3,000 deaths annually (Scallan et al., 2011), and about 72 billion dollars in economic losses (Scharff, 2012). In 2014, the Foodborne Diseases Active Surveillance Network (FoodNet) from 10 U.S. geographic areas reported 19,542 infections, 4,445 hospitalizations, and 71 deaths (Crim et al., 2015). Increased incidence of foodborne outbreaks and product recalls can be attributed to increased surveillance and reporting, modernization of food processing and agricultural practices, food consumption habits such as the desire for more natural preservative-free foods, increased at-risk populations, more accurate detection methods, antimicrobial resistance, and pathogens with improved adaptation and survivability upon exposure to stressors (Alvarez-Ordóñez et al., 2015, 2017; Begley and Hill, 2015; Bhunia, 2018).

Fascinating similarities exist between the food processing/preservation techniques and the host innate defense strategies. Therefore, the modern food processing practices (Van Boekel et al., 2010; Zhou et al., 2010; Davidson et al., 2013; Alvarez-Ordóñez et al., 2017), could “prime” microbes to be more invasive in the gut due to the ability of pathogens to withstand sub-lethal processing treatments and altered gene expression. Advanced food processing and preservation techniques are designed to reduce pathogen or toxin load or eliminate them from food; however, studies have suggested that food preservation techniques could create a sub-lethal environment (Capozzi et al., 2009). It is widely accepted that the sub-lethal treatments may trigger a bacterial stress response that results in changes in gene expression, leading to not only enhanced bacterial resistance to antimicrobials or preservation conditions but also enhanced pathogen survivability and virulence (Wesche et al., 2009; Spector and Kenyon, 2012; Verraes et al., 2013; Sun, 2014; NicAogáin and O’Byrne, 2016; Dawoud et al., 2017; Esbelin et al., 2018). Similarly, to overcome pathogenic microbial assault, a series of host defenses is strategically placed throughout the orogastric and intestinal tract (Fang et al., 2016). The host defense system includes enzymes, acidic pH, bile, resident microflora, antimicrobial peptides, mucus, and innate and adaptive immune responses, which help prevent or minimize pathogen colonization, invasion, and overall pathogenesis in a host (Sleator et al., 2009; Swaggerty et al., 2009; Garrett et al., 2010; Kamada et al., 2013; Bhunia, 2018).

**FIGURE 1:**
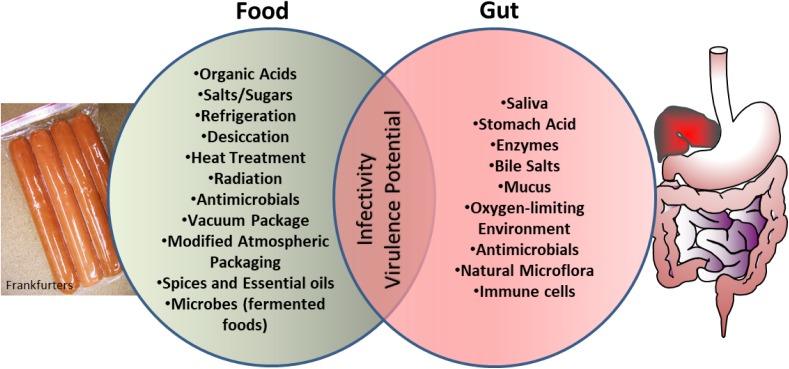
Schematics showing microbial exposure to shared stress environment between food processing conditions and human gut, which prime microbes for enhanced virulence and infectivity.

Another intriguing observation is that bacterial exposure to various food preservation, minimal processing, or sub-lethal treatment can change the nature and scale of antibiotic resistance in microbes (McMahon et al., 2007; Verraes et al., 2013) thus creating a situation where clinical management of these pathogens would be difficult. The current review explores the similarities that exist in the microenvironment of food preservation techniques and the host gastrointestinal tract aiding bacterial adaptation and readiness for increased infectivity (**Figure 1**).

## Food- and Host-Associated Bacterial Stressors

The objective of both host defense mechanisms and food preservation techniques is to eliminate or reduce microbial pathogens load; however, in many instances, the food processing treatments are sub-lethal and may trigger genes that are responsible for stress response. The stress response proteins help microbes to repair from injury and to cope with sub-lethal treatments. In addition, certain virulence genes are also upregulated under the stressful environment (Wesche et al., 2009; Dong and Schellhorn, 2010; Alvarez-Ordóñez et al., 2017). The food associated stressors are originated from a series of physical and chemical processing treatments (**Table 1**) that are employed to minimize or eliminate bacterial growth in food including drying (desiccation), heating, irradiation, ultrasound, sonication, electric pulse, hydrostatic pressure, acids, ethanol, salts and sugars, natural plant, or microbe-derived antimicrobial preservatives, modified atmospheric packaging, and oxidative treatments (Capozzi et al., 2009; Zhou et al., 2010; Davidson et al., 2013; Wang et al., 2016; Esbelin et al., 2018).

**Table 1:** Food processing/preservation conditions and host factors that may induce stress in pathogens.

Food preservation	Active components	Reference	Host factors	Active components	Reference
Heat treatments	Direct heat, electric pulse, microwave	Gabrić et al., 2018	Saliva	Enzymes (lysozyme, amylase, lipase)	Mandel, 1989
Cold treatments	Freezing, refrigeration	Tassou et al., 2010	Mucous	Mucin, enzymes, antimicrobial peptides, IgA	Hansson, 2012; Pelaseyed et al., 2014
Acidification	Acetic acid, citric acid, lactic acid, propionic acid, sorbic acid, gallic acid	Cruz-Romero et al., 2013; Wang et al., 2015	Acid	Hydrochloric acid (stomach), short chain fatty acids (SCFA, intestine)	Fang et al., 2016
Salts	Sodium chloride, phosphates, potassium, chloride, nitrate, nitrite	Zhou et al., 2010	Bile acids	Cholic acid, glycocholic acid, chenodeoxycholic acid, taurocholic acid, deoxycholic acid, lithocholic acid	Begley et al., 2005a; Payne et al., 2013; Joyce et al., 2014
Ethanol	Ethanol vapor	Capozzi et al., 2009; He et al., 2018			
Modified atmospheric packaging (MAP) or vacuum packaging	An optimal blend of oxygen, carbon dioxide and nitrogen or absence of oxygen	Caleb et al., 2013			
Irradiation	Microwave, X-ray, gamma-radiation, UV	Moosekian et al., 2012; Ahn et al., 2013	Enzymes	Trypsin, chymotrypsin, pepsin, cathepsin	Bhunia, 2018
Gas	Chlorine, ozone, nitrogen	Cortellino et al., 2015	Natural microflora	Proteobacteria, Bacteroidetes, Clostridiales	Becattini and Pamer, 2017
Antimicrobials	Lysozyme, lactoferrin, defensins, ovotransferrin, protamine, pleurocidin, bacteriocins,	Davidson et al., 2013	Antimicrobial peptides	Defensin, cryptdin	Brogden, 2005; Jäger et al., 2010
Spices and essential oils	Allicin, carvacrol, cinnamaldehyde, eugenol, geranial, thymol	Tiwari et al., 2009	Oxygen-limiting environment	Oxygen	Sewell et al., 2015; Wright et al., 2016
Desiccation	Water activity (Aw) below 0.85	Burgess et al., 2016; Esbelin et al., 2018	Immune cells	Macrophage, dendritic cells, NK cells	Garrett et al., 2010
Live microbes	Fermented products, lactic acid bacteria, yeast, acids, hydrogen peroxide	Ojha et al., 2015	Antibodies	IgA	Haneberg et al., 1994; Fang et al., 2016


The innate defense in the gastrointestinal tract is robust and generally effective in preventing foodborne pathogen interaction with the host. The gastrointestinal tract is over 23 ft long in an adult human. Multiple antimicrobial factors are present from mouth to rectum, that is, the entire length of the gastrointestinal tract (**Table 1**). Saliva in the mouth contains bacteriolytic enzymes (lysozyme), and gastric juice in the stomach contains hydrochloric acid and digestive enzymes, while the small and large intestine contain antimicrobial peptides (defensins, cathelicidin, cryptdin, elafin, etc.), bile, natural microflora, mucus, secretory IgA, oxygen-limiting environment, epithelial barrier, and submucosal immune cells (Brogden, 2005; Wesche et al., 2009; Garrett et al., 2010; Jäger et al., 2010; Sleator and Hill, 2010; Becattini and Pamer, 2017; Bhunia, 2018). Pathogens encounter multiple host-induced stresses in the intestine from acidic pH, nutrient limitation, low iron, oxidative and nitrosative stress, bile salts, free fatty acids, DNA damage, oxygen-limitation, and temperature in the gut (Louis and O’Byrne, 2010; Fang et al., 2016). If these factors fail to completely inactivate the microbes, they may be a source of stressors. Sub-lethal stressors, causing bacterial damage, elicit a bacterial stress response to initiate repair, or protect cells from stressors and increase the likelihood of survival.

## Common Food and the Host Factors That Affect Microbial Virulence

### Acid – A Major Food Preservative and a Disinfectant in Stomach

In the food system, microbial growth can be retarded or inhibited through acid shock from fermentation, preservatives (organic acids), and acid washes (Davidson et al., 2013). In addition, microbes may be exposed to the alkaline stress originating from the processing equipment, which is often treated with detergents or sanitizers (Capozzi et al., 2009; Wesche et al., 2009).

For microbial inactivation, organic acids such as lactic acid, acetic acid, citric acid, propionic acid, sorbic acid, and benzoic acids at a pH range of 4 to 6 are used. The antimicrobial action is mediated by both dissociated and undissociated ions depending on the final pH of the food matrix, affecting transmembrane proton motive force, inactivation of enzymes, cell injury, and cell death (Theron and Lues, 2007; Cruz-Romero et al., 2013; Wang et al., 2015). Furthermore, acidic pH can also cause damage to RNA and/or DNA (and subsequently alter protein synthesis), damage to membranes, spore coats, or through sequestration of cations regulating bacterial metabolic processes (Begley and Hill, 2015).

In the host gastric environment, an acidic pH 2 or lower is generally effective as a microbial barrier. There are, however, instances where bacteria can pass through the acidic barrier of the gut due to inconsistent acid secretions in the stomach, neutralization of acid by food or beverages or bacterial coping mechanisms or acid tolerance (Louis and O’Byrne, 2010). Acid-adapted bacteria such as *Salmonella* (Tsai and Ingham, 1997), *Escherichia coli* O157:H7 (Hsin-Yi and Chou, 2001; Foster, 2004), and *Listeria monocytogenes* (Gahan et al., 1996) can survive in the highly acidic environment (as low as pH 2.5). *E. coli, Shigella*, and *L. monocytogenes* use a glutamate decarboxylase (GAD) system to mitigate acidic pH (Cotter et al., 2005b). Besides, in *L. monocytogenes*, F_1_F_0_-ATPase and arginine deaminase, and in *L. innocua* a new type of ATP binding universal stress response protein (USP) also help in acid adaptation (Tremonte et al., 2016). In general, foodborne acid-adapted pathogens have a greater chance of survival during the orogastric passage and thus are more invasive than the non-acid adapted microbes.

Expression of acid shock proteins aids in bacterial coping mechanisms to survive acid conditions below a pH of two (Wesche et al., 2009; Dong and Schellhorn, 2010; Fang et al., 2016). Previous studies showed, for example, that during acid-shock, *Salmonella enterica* serovar Typhimurium (*S*. Typhimurium) induced expression of 60 genes related to stress resistance (Audia et al., 2001). In the course of acid exposure, sigma E (σ^E^) is highly activated and it increases bacterial survival in the acidified phagosomal vacuole in macrophages or dendritic cells (Muller et al., 2009). This allows *Salmonella* to evade the host immune system by avoiding oxidative stress in the vacuole (Crouch et al., 2005). Further studies show *S*. Typhimurium contains several two-component systems that are involved in virulence. Specifically, the EnvZ-OmpR is activated in response to acid stress, which enhances the type three-secretion system (TTSS) genes enhancing cellular replication (Fass and Groisman, 2009). CpxR-CpxA contributes to gut colonization and *Salmonella*-induced colitis (Fujimoto et al., 2018). In the presence of antimicrobial peptides, CpxR-CpxA-regulated genes are upregulated and contribute to the gut inflammation.

During exposure to gastric acid, enterohemorrhagic *E. coli* (EHEC) such as *E. coli* O157:H7 develop acid resistance through activation of an alternative sigma factor, RpoS (Barnett Foster, 2013). Research shows that acid-resistance can be acquired in the ruminant gut, leading to potential food contamination by more virulent acid- and cold-tolerant EHEC (Lin et al., 1996; Callaway et al., 2009). Acid-resistant EHEC also can tolerate the acidic environment (pH 1–3) in the human stomach. The acid resistance is governed by three genetic regulatory elements, RpoS; arginine decarboxylase (*adiA*) and its regulator, CysB; GAD (*gadA* or *gadB*); and γ-amino butyric acid antiporter (*gadC*) (Lim et al., 2010). Additionally, acid-resistant EHEC has been shown to alter gene expression patterns for adhesion and flagellar proteins, enhancing their ability to colonize the gut, although acid-resistance does not appear to induce Shiga-toxin mediated virulence (Barnett Foster, 2013). Furthermore, in EHEC, sensing of acyl-homoserine lactone appears to activate the transcription regulator SdiA which in turn upregulates locus of enterocyte effacement (LEE) pathogenicity island that encodes gene products required for attachment and effacement lesion and GAD promoting acid resistance (Hughes et al., 2010). Therefore, exposure to acids during food processing or storage enhances acid tolerance, thus ensuring bacterial safe transit through the stomach during foodborne infection. Likewise, contamination of food products with acid tolerant bacteria from meat animals helps bacterial resistance to acids used during food processing or preservation.

### Salt – A Common Food Preservative and a Natural Host Defense That Exerts Osmotic Stress

In food preservation, freeze-drying and storage in salt solution serve to eliminate bacteria or mitigate growth. Salts of sodium (NaCl), potassium (KCl), nitrate (NaNO_3_), or nitrite (NaNO_2_) are common food preservatives due to their exertion of osmotic stress on microbes. Osmotic stress, both a natural host defense and common in food preservation, mitigates bacterial growth and survival. Salt inhibits bacteria by disrupting the osmotic balance between the intracellular and cytoplasmic membrane (Wesche et al., 2009). Osmotic stress induces the filamentous appearance of bacterial pathogens such has been seen in *Salmonella, E. coli, Listeria*, and *Cronobacter* (Geng et al., 2003; Burgess et al., 2016). The endopeptidase that is required for cell division is downregulated during bacterial growth in the osmotic environment; hence, the cells are elongated (Burgess et al., 2016).

In the gut, bacteria are exposed to a hyperosmotic challenge, especially the bile salts, which is equivalent to 0.3 M NaCl and other ionic species (Chowdhury et al., 1996; Sleator and Hill, 2002). Therefore, osmoadaptation helps bacterial survival and increased virulence in a host. Various osmoregulatory systems become active in osmoadapted organisms, which include the production of osmoprotective compounds such as ProU in *Enterobacter*, ProP in *E. coli*, PutP in *Staphylococcus aureus*, and OpuC in *L. monocytogenes* (Sleator and Hill, 2002). Furthermore, many pathogenic bacteria commonly carry virulence genes and antibiotic resistance associated with ion transporters (Ganz and Nemeth, 2015; White et al., 2017). Harris et al. (2012) showed that *E. coli* O157:H7 exposed to 2% salt solution exhibited increased production of the Shiga toxin, which in part was due to activation of *recA* gene expression, indicating that osmotic stressors, similar to those that occur during meat processing, contribute to pathogen virulence.

Ethanol, starvation, and osmotic stress also increase microbial resistance to various antimicrobials (antibiotics) (Capozzi et al., 2009), induce biofilm formation, and persister traits (Poole, 2012). In persister cells, the gene loci, toxin–antitoxin (TA) is activated, thus antitoxin is degraded allowing the toxin to inhibit cellular processes such as DNA replication and protein translation, maintaining a non-replicative lifestyle (Helaine and Kugelberg, 2014; Page and Peti, 2016; Fisher et al., 2017). Persister phenotype helps bacteria to survive in an unfavorable condition such as nutrient limitation, extreme pH, and DNA damage by expressing high levels of intracellular guanosine tetraphosphate and guanosine pentaphosphate (p)ppGpp (Harms et al., 2016; Fisher et al., 2017). Many foodborne pathogens exhibit such trait which helps their persistence in food processing environment and in the host (Abee et al., 2016; Buchanan et al., 2017; Fisher et al., 2017; Wu et al., 2017). Osmotic stress increases microbial resistance to antibiotics and helps develop persister state, thus present a challenge for inactivation by sanitizers in the food system or by therapeutic antibiotics in humans.

### Antimicrobials, Proteins, and Enzymes Are Efficient Natural Biocides for Pathogens

Multiple antimicrobial proteins of prokaryotic or eukaryotic origin are being used or under investigation for potential use as food preservatives (**Table 1**; Garcia et al., 2010; Juneja et al., 2012). Bacteriocins such as nisin, pediocin, and reuterine are produced by lactic acid bacteria and are used or being considered for use in food preservation (Perez et al., 2014; Singh, 2018). Bacteriophages and endolysins are also considered for food preservation (Garcia et al., 2010; Schmelcher and Loessner, 2016; Goodridge et al., 2018). In addition, antimicrobial proteins from molds such as natamycin, tylosin, and polylysine are used in certain food products. Animal origin antimicrobials include chitosan, lysozyme, lactoferrin, lactoperoxidase, ovotransferrin, protamine, pleuricidin, and defensins have been considered for food preservation (Tiwari et al., 2009; Juneja et al., 2012; Davidson et al., 2013; Ray and Bhunia, 2014). Antimicrobial treatment may induce persister traits such as seen in *L. monocytogenes* after exposure to the antibiotics norfloxacin (Knudsen et al., 2013) or nisin (Wu et al., 2017). Likewise, *Salmonella, E. coli, Staphylococcus*, and others also exhibit persister phenotype after exposure to antimicrobials, which helps bacterial survival (Helaine and Kugelberg, 2014; Fisher et al., 2017). Pathogens in persister state in food can bloom in a host after consumption and can cause disease (Lewis, 2007).

The plant-derived phenolic compounds as secondary metabolites are originated from the metabolism of phenolic acids, flavonoids, stilbenes, lignans, and tannins in the gut. These phenolic compounds also exert an antimicrobial effect on pathogens (Selma et al., 2009) and may induce stress in pathogens.

The host-derived metabolic compounds, such as enzymes, proteins, and immunoglobulins can also exert an antimicrobial effect. Host enzymes such as lysozyme or phospholipase disrupt microbial cell membranes (Mandel, 1989). Host immune proteins, such as IgA or innate immune proteins lipocalin-2, inactivate microbes. Lipocalin-2 production is stimulated by the host inflammatory response and binds siderophores, thus limiting iron uptake and preventing microbial growth (Flo et al., 2004; Raffatellu et al., 2009). Commensal microbes can protect the host from pathogenic microbes through competitive exclusion and production of antimicrobial peptides (bacteriocins) although some pathogenic microbes can evade such a barrier (Cotter et al., 2005a; Hibbing et al., 2010; Becattini and Pamer, 2017).

### Modified Atmospheric Packaging or Reduced Oxygen-Levels Is a Common Source of Microbial Stress in Both Food Products and Human Gut

Modified atmospheric packaging (MAP) is a minimal processing practice to prevent pathogen growth. In MAP, an optimal blend of oxygen, carbon dioxide, and nitrogen is present within a high barrier or permeable package (Caleb et al., 2013). During MAP and vacuum packaging of food products, oxygen level is minimal or absent, which in turn limits oxygen availability for aerobic biochemical processes and in some cases this induces a bacterial stress response in aerobic bacteria (Wesche et al., 2009; Poole, 2012). In the host gastrointestinal tract, the oxygen level gradually decreases from proximal small intestine to distal large intestine and provides a favorable growth environment for anaerobic pathogens. Since many foodborne pathogens are aerobic or facultative anaerobe thus they remain in close proximity to the mucosal epithelial cells where they gain access to oxygen from host cells during colonization of the gut. For example, *Salmonella* expresses type 1 fimbriae that facilitate the bacterial invasion of oxygen-containing host cells and the fimbriae expression is high during aerobic growth but not in an anaerobic environment (Ernst et al., 1990; Hakalehto et al., 2007). Furthermore, for survival under anaerobic environment, pathogens also employ different strategies. In the course of *Salmonella* infection, epithelial cells generate reactive oxygen species (ROS), which react with thiosulfate produced by the gut microbiota and convert it into tetrathionate, a terminal electron acceptor, which is used by the bacterium for growth (Winter et al., 2010; Behnsen et al., 2015).

**FIGURE 2:**
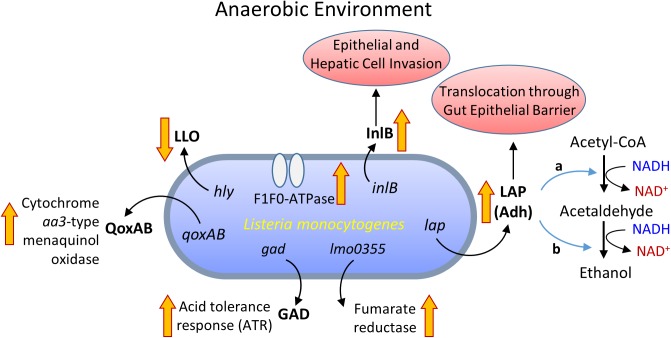
Metabolic activity and virulence gene expression in *Listeria monocytogenes* under anaerobic growth environment. LLO, listeriolysin O; InlB, internalin B; QoxAB, cytochrome aa3-type menaquinol oxidase; GAD, glutamate decarboxylase; LAP, Listeria adhesion protein or alcohol acetaldehyde dehydrogenase (Adh) consists of bifunctional acetaldehyde dehydrogenase (a) and alcohol dehydrogenase (b).

*Listeria monocytogenes* was shown to up-regulate 28 genes under anaerobic condition (Mueller-Herbst et al., 2014), of these, *lmo0355* encoding fumarate reductase expression was high (**Figure 2**). In addition, generation of a proton motive force via F_1_F_0_-ATPase was essential for growth under the anaerobic environment (Mueller-Herbst et al., 2014). While, in the aerobic environment, a redox-responsive transcription factor, *spxA1*, is necessary for the growth of *L. monocytogenes* (Whiteley et al., 2017). In addition, *L. monocytogenes* uses a cytochrome *bd*-type (CydAB) terminal oxidase for respiration under aerobic environment while a cytochrome aa3-type menaquinol oxidase (QoxAB) for respiration under reduced oxygen levels possibly during host infection (Corbett et al., 2017). Under the anaerobic condition in the presence of central carbon metabolism intermediates, such as acetate, citrate, fumarate, pyruvate, lactate, and succinate, *L. monocytogenes* expresses reduced listeriolysin O (LLO) but the higher invasion of cultured cell lines (Wallace et al., 2017). Anaerobic growth also promotes enhanced *L. monocytogenes* adhesion and invasion (Andersen et al., 2007; Burkholder and Bhunia, 2009). Increased cell invasion could be attributed to increased expression of internalin B (InlB) during anaerobic growth, involved in adhesion and invasion of mucosal epithelial cells and hepatic cells (Lindén et al., 2008).

*Listeria monocytogenes* also expressed high levels of *Listeria* adhesion protein (LAP) also known as alcohol acetaldehyde dehydrogenase (Adh), which catalyzes the conversion of acetyl-CoA to acetaldehyde and from acetaldehyde to ethanol during growth under the anaerobic environment (**Figure 2**; Jagadeesan et al., 2010; Mueller-Herbst et al., 2014). The LAP is responsible for *L. monocytogenes* adhesion to host intestinal epithelial cells (Pandiripally et al., 1999; Jaradat et al., 2003; Wampler et al., 2004) and bacterial paracellular translocation across the gut intestinal epithelial barrier (Burkholder and Bhunia, 2010, 2013; Kim and Bhunia, 2013; Drolia et al., 2018). Under anaerobic condition, *in vitro, L. monocytogenes* expresses a high level of LAP and induces increased adhesion to Caco-2 cells and increased extra-intestinal dissemination in mice (Burkholder et al., 2009). This implies that oxygen-limiting vacuum-packaged food likely helps the bacterium to adapt and invade host upon entering the host gastrointestinal tract through contaminated food (Andersen et al., 2007; Burkholder et al., 2009; **Figure 2**). The oxygen-limiting condition also increases acid tolerance and aids *L. monocytogenes* transit from the stomach to the intestine (Sewell et al., 2015), possibly due to upregulation of GAD (Gahan and Hill, 2014).

In EHEC, low oxygen can stimulate expression of virulence factors such as Sfp fimbriae which enhances bacterial colonization in the gut (Müsken et al., 2008; Barnett Foster, 2013) through upregulation of TTSS and EspA (Schüller and Phillips, 2010). Likewise, *S. flexneri*, under anaerobic environment, showed differential upregulation of 528 genes, of which 228 genes were influenced by fumarate and nitrate reduction regulator (FNR) (Vergara-Irigaray et al., 2014). Furthermore, genes encoding TTSS, required for bacterial invasion of host cells and pathogenesis were also upregulated under anaerobiosis (Vergara-Irigaray et al., 2014). *Vibrio cholerae* under oxygen-limiting condition also induces cholera toxin (CT), toxin-coregulated pili (TCP), and AphB, a transcriptional activator of TcpP expression in the host (Krishnan et al., 2004; Liu et al., 2011). This bacterium may use a thiol-based switch system to sense intestinal environment for virulence protein expression (Liu et al., 2011).

*Campylobacter* is a microaerophilic pathogen with a strict requirement for oxygen (O_2_), hydrogen (H_2_), and carbon dioxide (CO_2_); however, under anaerobic condition, *Campylobacter* expresses several putative virulence factors (Lee et al., 2014) for increased motility and epithelial cell invasion (Mills et al., 2012).

In case of anaerobic pathogens such as *Clostridium botulinum, C. perfringens*, and *C. difficile* growth is supported by the oxygen-deficient environment of food. Upon entry into the host through contaminated food, they can find a niche for colonization in the host intestine (Rossetto et al., 2014; Lessa et al., 2015; Abt et al., 2016; Freedman et al., 2016). Innate host defense may include the release of the stress hormone, epinephrine, and norepinephrine, which severely affect virulence gene expression and iron acquisition and quorum sensing abilities of anaerobic pathogens (Boyanova, 2017). Overall, the microbes that are adapted to the oxygen-limiting environment of the food are well equipped to not only survive in the human intestine but also show enhanced colonization, invasion, and pathogenesis.

### Reactive Oxygen Species-Induced Oxidative Stress Is Harmful to Aerobic Bacteria

Reactive oxygen species include hydrogen peroxide (H_2_O_2_) and superoxide (${\text{O}}_{2}^{-}$), which exert oxidative stress in microbes that grow aerobically. ROS damages DNA, membranes, and proteins (Imlay, 2003). Bacteria encounter oxidative stress in both food/food processing environment, such as H_2_O_2_-based disinfectants used for sanitization of processing equipment or food contact/non-contact surfaces. ROS are also present in immune cells, especially in neutrophils and macrophages in the host. *L. monocytogenes* express 2-cys peroxiredoxin (*prx*) to cope with increasing concentration of H_2_O_2_ *in vitro* but not in a mouse model where a *prx* mutant strain did not show growth defect in mouse liver or spleen (Kim et al., 2007). However, this contrasts with another study (Dons et al., 2014) where authors observed reduced virulence of a *prx* mutant in mice. In addition, H_2_O_2_ induced stress also results in increased transcription of σ^B^ and *kat* at 37°C but not at 20°C and *L. monocytogenes* exhibits higher resistance to H_2_O_2_ at 20°C and petite colony phenotype (Ochiai et al., 2017).

*Campylobacter jejuni* also regulates oxidative stress defense for survival (Kim et al., 2015). To neutralize superoxide mediated oxidative stress or cell damage, many pathogens including *E. coli* (Carlioz and Touati, 1986), *C. jejuni* (Pesci et al., 1994; Purdy and Park, 1994), *S*. Typhimurium (Fang et al., 1999), *S. flexneri* (Franzon et al., 1990), *S. aureus* (Kanafani and Martin, 1985), and *L. monocytogenes* (Vasconcelos and Deneer, 1994) express superoxide dismutase (SOD) (Chiang and Schellhorn, 2012). Furthermore, the multidrug efflux system is over-expressed in microbes during oxidative stress; thus they exhibit resistance to multiple antimicrobials including antibiotics (Poole, 2012). In addition, oxidative stress can induce biofilm formation and promote persister trait (Zhang, 2014).

### Bile, a Major Microbial Inhibitor in the Intestine

Another significant host challenge to microbes is exposure to bile in the intestine. Bile contains acids (deoxycholic acid and lithocholic acid), salts, and enzymes that, at least in part, are responsible for the cellular breakdown of microbes through membrane and DNA damage (Begley et al., 2005a; Merritt and Donaldson, 2009; Sistrunk et al., 2016). As expected, there is a degree of microbial resistance to bile components (Gunn, 2000; Louis and O’Byrne, 2010). Specifically, the lipopolysaccharide (LPS) structure in the outer membrane in Gram-negative bacteria and the presence of specific porins protect cell membrane from bile. Studies have shown that Gram-negative pathogens expressing the OmpF porin contain a greater degree of tolerance to bile components. Furthermore, expression of the AcrAB efflux pump on some pathogens such as in *E. coli* and *S. flexneri* may contribute to bile resistance (Thanassi et al., 1997; Nickerson et al., 2017). In the presence of bile, *S. flexneri* also forms a biofilm and induces genes responsible for multidrug resistance and virulence (Nickerson et al., 2017). An additional coping mechanism to bile, common in gut microflora, is the presence of bile salt hydrolase (BSH) (Jones et al., 2008; Joyce et al., 2014).

Studies have shown that EHEC modulate virulence factor expression in the presence of bile during passage through gut (Barnett Foster, 2013). With bile as an environmental cue, EHEC and *Shigella* can up-regulate AcrAB efflux pump and lipid A modification pathway, allowing for improved membrane integrity (Rosenberg et al., 2003; Kus et al., 2011; Nickerson et al., 2017). *L. monocytogenes* also expresses BSH and BilE (bile salt exclusion protein), regulated by sigma B (σ^B^) to neutralize or exclude the effect of bile thus survive in the intestine (Begley et al., 2005b; Sleator et al., 2005; Sleator and Hill, 2010). In the presence of bile acid deoxycholate, *C. jejuni* expresses virulence genes including *Campylobacter* invasion antigen (*ciaB*) and other virulence genes for the enhanced invasion of epithelial cells (Malik-Kale et al., 2008; Novik et al., 2010). In general, enteric pathogens have developed elegant strategies to survive in the presence of bile in the intestine, and capable of causing serious illness. However, if food were contaminated with bile resistant microbes, control would be much more difficult due to their cross protection against other inhibitors.

## Repair, Adaptation, Subsequent Cross-Protection Against Stressors, and Virulence

### Quorum Sensing Is an Important Mechanism for Microbial Survival Under Stress Environment

Microbial byproducts, such as butyrate and the quorum-signaling molecule autoinducer-3 (AI-3) may alter bacterial virulence genes (Barnett Foster, 2013). Previous research suggests that the butyrate concentration may affect adhesion properties and upregulation of siderophores (Barnett Foster, 2013). Secretion of AI-3 has been shown to increase EHEC motility through flagellar biosynthesis (Clarke et al., 2006). The two-component PhoQ/PhoP system in *Salmonella* is activated in the presence of antimicrobial peptides, rendering protection and further activation of the PmrB/PmrA that influences membrane structure during exposure to a stressor (Gunn, 2000).

Although not fully understood, the host gut signaling hormones epinephrine and norepinephrine play a role in the induction of EHEC virulence genes responsible for chemotaxis, biofilm formation and bacterial adhesion to enterocytes (Bansal et al., 2007; Moreira and Sperandio, 2010; Barnett Foster, 2013). EHEC receptor kinase sensor QseE is, for example, sensitive to epinephrine, and its activation enhances EHEC colonization (Moreira and Sperandio, 2010; Sperandio and Nguyen, 2012). QseC histidine kinase sensor helps *Salmonella* to sense AI-3, epinephrine or norepinephrine for increased motility, invasion of epithelial cells and survival inside macrophages (Moreira et al., 2010). In *Campylobacter*, norepinephrine also increases bacterial growth rate, motility, cell invasion, and disruption of the epithelial tight junction (Cogan et al., 2007). These indicate that stress and consequent infection may dictate the severity of foodborne diseases.

### Sigma Factors Are Fundamental Stress Regulators

Bacteria have evolved to have stressor coping mechanisms, namely, the ability to sense the environment in the gut (or preserved food) and elicit changes in gene expression to cope with specific stressors (NicAogáin and O’Byrne, 2016). Two mechanisms are used by microbes to alter gene and protein expression during exposure to stressors, the signal transduction system and alternative sigma factors such as σ^B^, σ^S^, σ^E^, σ^F^, σ^N^, RpoE, RpoH, and RpoS (Kazmierczak et al., 2005). The signal transduction system is a coping mechanism in which a membrane-associated sensor is phosphorylated due to an external signal. A response regulator is subsequently activated, which plays a major role in the secretion of cationic peptides by the innate immune system (Louis and O’Byrne, 2010). Generally, the response of stress stimuli in damaged bacteria leads to expression of factors to guide RNA polymerase for inducing repair or to express proteins required for survival (Wesche et al., 2009). Alternative sigma factors play an important role in promoter recognition and production of cellular proteins to support virulence mechanisms (Kazmierczak et al., 2005). σ^B^ plays a major role in exposure to acids, salts, and bile (Kazmierczak et al., 2005; Louis and O’Byrne, 2010). In response to a stress, such as low pH, the stressosome is activated and the phosphatase RsbU, which subsequently allows RsbV anti-anti-sigma factor to bind to anti-sigma factor RsbW rendering the σ^B^ free to guide the RNA polymerase.

Another alternative sigma factor, RpoS, plays a role in *Salmonella* and *E. coli* acid tolerance and virulence (Hengge-Aronis and Storz, 2000; Foster, 2004; Dong and Schellhorn, 2010). Stress-induced activation of RpoS activates the *esp* genes in pathogenic *E. coli* essential for attachment and effacement lesion during pathogenesis (Laaberki et al., 2006). Furthermore, activation of RpoS helps *V. cholera* to evade host immune system allowing the pathogen to be localized in the intestinal lumen and consequent shedding into the environment (Conner et al., 2016). The molecular basis of the RpoS regulator includes stress-cue activation of anti-adaptor proteins, which release the protein RssB that forms a RssB-RpoS complex. This complex is further degraded by the ClpXP protease rendering a free RpoS that guides the RNA polymerase (Jaishankar and Srivastava, 2017).

σ^E^ is another alternative sigma factor activated by periplasmic stress leading to the activation of genes involved in degradation and refolding of damaged proteins (Rhodius and Mutalik, 2010). Heat, cold, and ethanol stressors have been shown to activate σ^E^ (Rowley et al., 2006). Activation of σ^E^ in Gram-negative bacteria is initiated by stress perception. Specifically, unfolded proteins (generated from oxidative stress for example) interact with membrane-associated proteases (DegS and RseP) and degrade RseA releasing σ^E^. The free RseA- σ^E^ complex is then tagged by SspB and subsequently degraded by protease ClpXP allowing for σ^E^ to interact with the RNA polymerase, guiding expression of proteins to enhance protein synthesis (Louis and O’Byrne, 2010; Jaishankar and Srivastava, 2017).

### Adaptation and Cross-Protection Against Stressors

Another significant factor to consider with environmental induction of pathogen virulence is cross-protection, that is, resistance to multiple environmental stressors derived from bacterial exposure to a specific environmental stressor (Capozzi et al., 2009; Alvarez-Ordóñez et al., 2015, 2017). For example, several studies have shown that starvation stress induces heat tolerance and resistance to oxidative stress in pathogenic *E. coli* and *Serratia marcescens* (Jenkins et al., 1988; Leenanon and Drake, 2001; Pittman et al., 2015).

Adaptation of *S*. Typhimurium to acid stress is linked to cross-protection against salt and oxidative stress (Leyer and Johnson, 1993). Likewise, acid-adapted *Salmonella, E. coli* O157:H7 and *L. monocytogenes* showed increased resistance to heat (Mazzotta, 2001; Haberbeck et al., 2017). Conversely, bacterial growth at higher temperatures can also evoke cross-protection against other lethal treatments. For example, *S*. Enteritidis, grown at 37°C showed increased cell membrane fluidity, acid resistance, and RpoS expression; while at 42°C, bacteria showed increased heat resistance and RpoH expression, and decreased RpoS expression (Yang et al., 2014). At 42°C, *S*. Enteritidis also showed induction of virulence-related genes, *spvR, hilA*, and *avrA* (Yang et al., 2014). In response to heat shock, the presence of damaged or denatured proteins is thought to be stimuli for activation of alternative sigma factors and subsequent activation of heat shock proteins that are involved in protein repair (Wesche et al., 2009). Kobayashi et al. (2005) showed that heat treatment (55°C for 15 min) resulted in up-regulation of 19 heat-inducible and 12 oxidative-stress and DNA damage-inducible genes in *S*. Enteritidis. In a separate study, Sirsat et al. (2011) showed that sublethal heat treatment (42°C for 10 or 30 min) in *S*. Typhimurium induced genes encoded in SPI-2 and SPI-5 especially transcription of genes encoding fimbriae and Rpo regulons. Sub-lethal heat stressed *S*. Typhimurium also showed increased adhesion and invasion of intestinal enterocyte-like Caco-2 cells and virulence (Burkholder and Bhunia, 2009; Sirsat et al., 2011; Dawoud et al., 2017).

*Escherichia coli* O157:H7 exposed to progressively intensifying milder heat (54–60°C) treatment displayed higher resistance to high hydrostatic pressure possibly by activating RpoS and RpoH (Gayán et al., 2016). Interestingly, in *C. botulinum*, exposure to prolonged heat stress (45°C), heat shock genes and members of the SOS regulons were activated while genes encoding neurotoxin (*botA*) synthesis was downregulated (Selby et al., 2017). In *L. monocytogenes*, growth at 37°C induced its adaptation to acid (Shen et al., 2014), salts (6% NaCl), and hydrogen peroxide (Bergholz et al., 2012).

In the presence of low temperatures, cold stress proteins have been shown to play important roles in microbial viability (example, *L. monocytogenes, E. coli*, and *Bacillus cereus*) (Wesche et al., 2009; Saldivar et al., 2018). Subsequent to cellular sensing of abnormally cold temperatures, alternative sigma factors are induced, leading to expression of cold-shock proteins that aid in membrane fluidity, protein folding, and nutrient uptake (Wouters et al., 2000). Interestingly, pre-exposure to cold stress also increases bacterial cross protection against osmotic stress. *L. monocytogenes* exposed to refrigerated temperature showed increased survival in 3% NaCl, and showed higher expression of proteins required for maintenance of cell wall and cellular processes such as osmolyte transporters, amino acid metabolism, and lipid biosynthesis (Pittman et al., 2014).

Pre-exposure to organic acid salts (potassium lactate), *L. monocytogenes* exhibited resistance against food antimicrobials such as nisin, lauric arginate, and 𝜖-polylysine. This cross-protection involves activation of two-component response-regulator, VirRS (Kang et al., 2015). Similarly, in the presence of NaCl (6%), *L. monocytogenes* exhibited resistance against nisin (Bergholz et al., 2013). In a food product such as in soft cheese environment (containing acid and salts), *L. monocytogenes* displayed cross-protection against antimicrobials thus may help the bacterium to overcome antimicrobials encountered in the host intestine (Melo et al., 2015).

Growth under oxygen-limiting environment also increases bacterial acid tolerance (Sewell et al., 2015) and resistant to bile salts (Payne et al., 2013; Wright et al., 2016), and survival in the gastrointestinal tract such was seen in *L. monocytogenes*. Conversely, sub-lethal exposure to antibiotics renders *L. monocytogenes* to shift to anaerobic metabolism and the bacterium made reduced levels of virulence proteins including LLO, InlB, and LAP; however, exhibited increased bacterial tolerance to multiple other antibiotics (Knudsen et al., 2016; Zhu et al., 2018).

Adaptation to ethanol caused *S*. Enteritidis to be more resistant to acid and showed upregulation of acid tolerant regulator, RpoS (He et al., 2018). Similarly, prolonged exposure (24 h) of acidic stress protected *S*. *aureus* against non-thermal plasma treatment exhibiting reduced cell membrane damage, membrane potential, and intracellular enzyme activity (Liao et al., 2018). Altogether, cross-protection must be considered a significant bacterial coping mechanism that may have broad implications in regards to pathogen survival in the gut, resistance to antimicrobials and increased virulence.

## Food Products and Microbial Virulence

Studies have shown that food environment significantly affects microbial pathogenesis (Ponder, 2017) and antimicrobial resistance depending on the specific stressors (discussed above) microbes encounter in a food (**Table 2**). *L. monocytogenes* cultured in ready-to-eat (RTE) meat matrices appeared to have increased invasiveness in a cell culture J774A.1 (Larsen et al., 2010; Lin et al., 2010) or in a mouse (Mahoney and Henriksson, 2003) model. In fresh cut melon inoculated with *L. monocytogenes*, and stored at 10°C for 2 days, the bacterium was highly invasive to Caco-2 cells (Colás-Medà et al., 2017). Similarly, *L. monocytogenes* showed increased invasiveness to Caco-2 cells when stored in pasteurized milk than raw milk at 4°C (Pricope-Ciolacu et al., 2013). *L. monocytogenes* obtained from a fermented sausage and cured cooked ham stored under 10°C for up to 4 weeks were also highly invasive to Caco-2 cells (Larsen et al., 2010). Furthermore, *L. monocytogenes* grown in meat juice differentially expressed high levels of virulence genes such as *gad*, σ^B^, *sod*, and *inlA* (Rantsiou et al., 2012). Likewise, in liver pâté, *prfA*, σ^B^, and *inlA* were upregulated (Olesen et al., 2010) and in salami, acidic (σ^B^ and *lmo0669*) and osmotic (*gbuA* and *lmo1421*) stress-related genes were upregulated (Mataragas et al., 2015).

**Table 2:** Effect of food products on virulence potential of foodborne pathogens.

Pathogen	Food	Virulence potential	Reference
*Bacillus* spp.	Infant formula	Increased enterotoxin production and cytotoxicity to Caco-2, and HEp-2 cell lines	Rowan et al., 2001
*Listeria monocytogenes*	Ready-to-eat meat	Increased invasion to macrophage cell line (J774A.1)	Lin et al., 2010
	Deli meat in modified atmosphere packaging	Increased invasion to Caco-2 and INT-407 cell lines	Larsen et al., 2010
	Ground meat and fermented sausage	Transcriptome array showed upregulation of *gad*, σ^B^, *sod*, and *inlA* genes	Rantsiou et al., 2012
	Liver pâté	Upregulation of *prfA*, σ^B^, and *inlA*	Olesen et al., 2010
	Salami	Acidic (σ^B^ and *lmo0669*) and osmotic (*gbuA* and *lmo1421*) stress-related genes were upregulated	Mataragas et al., 2015
	Pear and Melon	Increased adhesion and invasion of Caco-2 cells after 2 days of storage	Colás-Medà et al., 2017
	Milk	Increased invasiveness to Caco-2 cells when stored in pasteurized milk than raw milk at 4°C	Pricope-Ciolacu et al., 2013
*Staphylococcus aureus*	Cheese, ham, sausage	Increased production of enterotoxins	Schelin et al., 2011
	Cheese (*Lactococcus lactis*)	Enterotoxin genes (*sea, sel*, and *she*) were upregulated while stress regulators (σ^B^ and *agr*) were downregulated	Cretenet et al., 2011
*Salmonella*	Sequential incubation into the soil, lettuce and cut lettuce stored under modified atmosphere conditions	Increased survival in simulated gastric and intestinal fluid, but reduced adhesion and invasion of Caco-2 cells	Oliveira et al., 2011
	Parsley	Production of curli and cellulose on parsley plants	Lapidot and Yaron, 2009


*Bacillus* species grown in reconstituted infant formula containing glucose exhibited increased expression of enterotoxin production and cytotoxicity when tested on Caco-2 and HEp-2 cell lines (Rowan et al., 2001). *S. aureus* also showed increased production of enterotoxin in various food products including cheese, ham, and sausage containing high levels of salts (Schelin et al., 2011). In cheese under the acidified environment (containing *Lactococcus lactis*), genes for enterotoxin *sea, sel*, and *seh*, and the stress response genes, *dnaK, sodA*, and others were upregulated while the stress regulator, σ^B^, and *agr* were downregulated (Cretenet et al., 2011). *S*. Typhimurium grown sequentially in soil, lettuce and cut lettuce and stored under modified atmosphere (MAP) conditions showed increased survival in simulated gastric and intestinal juice but exhibited reduced adhesion and invasion of Caco-2 cells *in vitro* suggesting the food matrix or environmental factors may have differential effect on *Salmonella* virulence when analyzed *in vitro* (Oliveira et al., 2011).

## Conclusion and the Future Perspectives

Natural inhibitors exist in the host gastrointestinal tract to eliminate pathogenic bacteria including acidic pH, low oxygen levels, changes in osmolarity, antimicrobials, and ROS and oxidative stress. Similarly, food preservation and sanitation techniques are employed to eliminate foodborne pathogens. Bacterial stress or injury happens when chemical or physical treatment results in a sub-lethal damage to the microorganisms. The general bacterial response to sub-lethal stress triggers gene expression through activation of signal transduction systems and alternative sigma factors. Through environmental cues, alternative sigma factors are activated that guides RNA polymerase, gene reprogramming, and production of proteins to support bacterial coping with a stress event. Therefore, the stressed bacteria have improved survival strategy such as biofilm formation, resistance to antimicrobials, and persister state, which under favorable condition can bloom and show increased virulence. Furthermore, exposure to a primary stressor may lead to the onset of cross-resistance (or adaptation) to multiple secondary stressors, antibiotic resistance, and increased virulence. Due to the similarity of stressors between the food preservation treatments and the gut innate defense, and their impact on pathogen physiology and behavior, it is a grim reality that the food processing treatments could prime the microbial pathogens for enhanced survival and infectivity in a host (Lin et al., 1996; Lim et al., 2010; Alvarez-Ordóñez et al., 2015, 2017; Buchanan et al., 2017; Fisher et al., 2017). Therefore, the modern food processing and production practices employed today, ironically are a curse in disguise, and possibly a major contributing factor for the emergence of increased incidence of foodborne illnesses, outbreaks, and fatality around the globe.

The current trend is to use minimal processing or hurdle approach to reduce pathogen load in foods; therefore, we need to have a greater understanding of the sub-lethal effect of such processing treatments on microorganisms, especially their physiology, behavior or pathogenesis. Furthermore, new and emerging technologies such as ultra violet (UV) rays (Kim et al., 2017), X-ray (Mahmoud et al., 2016), cold plasma (Niemira, 2012; Lu et al., 2014), and bacteriophages (Schmelcher and Loessner, 2016; Goodridge et al., 2018; Shahin and Bouzari, 2018) are attractive and are being used in industrial settings. These processing methods directly affect genetic elements of microbes; however, it is unknown, the impact of sub-lethal treatment or prolonged exposure to microbial physiology, virulence, and infectivity. UV, X-rays, and bacteriophages are known to cause gene deletion or insertion (mutation) in microbes, and survival and spread of such microbes could pose a grave danger to our food safety and public health management practices.

Furthermore, there seems to be a scarcity of information linking pathogen virulence in humans to sub-lethal stress exposure in the meat or companion animals. Several interesting aspects that need future investigations such as pathogenesis and virulence gene expression in pathogens in a meat animal model (for example swine) where the animal has experienced multiple stressors (for example changes in nutrition, transportation, or management procedures). A better understanding of the relationship between food production and preservation techniques and induction of pathogen virulence may lead to techniques and procedures to reduce foodborne illnesses.

## Author Contributions

NH and AB designed the study, reviewed literature, and relevant articles and wrote the manuscript. AB made the figures and tables. All authors read and approved the manuscript.

## Conflict of Interest Statement

The authors declare that the research was conducted in the absence of any commercial or financial relationships that could be construed as a potential conflict of interest.
